# Antimicrobial, Cytotoxic, Phytotoxic and Antioxidant Potential of *Heliotropium strigosum* Willd.

**DOI:** 10.3390/medicines3030020

**Published:** 2016-07-28

**Authors:** Muhammad Khurm, Bashir A. Chaudhry, Muhammad Uzair, Khalid H. Janbaz

**Affiliations:** 1 Faculty of Pharmacy, Natural Product Chemistry Unit, Bahauddin Zakariya University, Multan 60800, Pakistan; drbashirahmadch@bzu.edu.pk (B.A.C.); muhammaduzair@bzu.edu.pk (M.U.); 2 Akson College of Pharmacy, Mirpur University of Science and Technology, Mirpur 10250, Pakistan; KHjanbaz@hotmail.com

**Keywords:** *Heliotropium strigosum*, antimicrobial activity, cytotoxicity, phytotoxicity, antioxidant activity, *Boraginaceae* family

## Abstract

**Background:** *Heliotropium strigosum* Willd. (Chitiphal) is a medicinally important herb that belongs to the *Boraginaceae* family. Traditionally, this plant was used in the medication therapy of various ailments in different populations of the world. The aim of the study is to probe the therapeutic aspects of *H. strigosum* described in the traditional folklore history of medicines. **Methods:** In the present study, the dichloromethane crude extract of this plant was screened to explore the antimicrobial, cytotoxic, phytotoxic and antioxidant potential of *H. strigosum*. For antibacterial, antifungal and antioxidant activities, microplate alamar blue assay (MABA), agar tube dilution method and diphenyl picryl hydrazine (DPPH) radical-scavenging assay were used, respectively. The cytotoxic and phytotoxic potential were demonstrated by using brine shrimp lethality bioassay and *Lemna minor* assay. **Results:** The crude extract displayed positive cytotoxic activity in the brine shrimp lethality assay, with 23 of 30 shrimps dying at the concentration of 1000 µg/mL. It also showed moderate phytotoxic potential with percent inhibition of 50% at the concentration of 1000 µg/mL. The crude extract exhibited no significant antibacterial activity against *Staphylococcus aureus*, *Shigella flexneri*, *Escherichia coli* and *Pseudomonas aeruginosa*. Non-significant antifungal and radical scavenging activity was also shown by the dichloromethane crude extract. **Conclusion:** It is recommended that scientists focus on the identification and isolation of beneficial bioactive constituents with the help of advanced scientific methodologies that seems to be helpful in the synthesis of new therapeutic agents of desired interest.

## 1. Introduction

For the past few decades, the importance of medicinal plants for treating various infections has been tremendously increased because of the fact that a large number of people belonging to different populations depend upon the usage of phytomedicines due to the unavailability of primary healthcare facilities [1]. According to World Health Organization reports on phytomedicines, more than 25% of drugs which have been prescribed in recent years are obtained from different plant sources [2]. The family *Boraginaceae* is comprised of 100 genera and about 2000 species. The plants of this family are widely distributed in temperate, especially Mediterranean and tropical, regions. In Pakistan, this family is represented by 32 genera and 135 species. Moreover, some species, namely *Cordia*, *Echium* and *Anchusa* are cultivated [3]. *Heliotropium*, *Cordia*, *Arnebia*, *Martensia* and *Trichodesma* are the important genera of the *Boraginaceae* family. Fruits of *Cordia* are used as diaphoretic and sometimes as astringent [4]. The leaves and roots of *Trichodesma indicum* are effective against snake bites and urinary diseases and are used as a diuretic. The roots of this plant are also applied as a paste on swellings and joints and are used in dysentery in children [5]. Today, *Alkanna* (*Alkanna tinctoria*) root is used almost exclusively as a cosmetic dye. Orally, it has been used for diarrhea and gastric ulcers. Traditionally, *Alkanna* root has been used topically to treat skin wounds and diseases [6].

*Heliotropium* is one of the most complex and largest genera of the family *Boraginaceae*. In tropical and temperate regions, it was represented by 270–275 species while in Pakistan 23 species of this genus are present [3]. In folk medicinal history, species of genus *Heliotropium* have attained noticeable pharmacological importance. In Somalia, the pulp of roots of *H. aegyptiacum* was considered to be effective against scorpion stings and snake bites [7]. In India, the paste of the leaves of *H. indicum* was used for rheumatism [8]. In Mauritius, the decoction of whole plant of *H. amplexicaule* was used in the therapeutic management of coughs and fevers [9]. The variety of traditional medicinal uses of *H. strigosum* made it distinguishable among other species of genus *Heliotropium*. The powder and decoction of the whole plant material of *H. strigosum* has been used in the medication therapy of rheumatic arthritis and jaundice and it is also used as a blood purifier [10]. The paste of roots of this plant is used for healing wounds [11]. For the curing of snake bites, gum boils, eye sores and nettle stings, the juice of this whole plant is administered [12]. This juice has also been used as diuretic and demonstrated some laxative effect [13]. In the current study, the dichloromethane crude extract of *H. strigosum* was examined for different biological activities, which could be related to the potential therapeutic value of this plant as prescribed in traditional medicine.

## 2. Experimental Section

### 2.1. Materials and Methods

The current study was conducted in the natural product chemistry laboratory, Faculty of Pharmacy, Bahauddin Zakariya University, new campus Multan and International Centre for Chemical and Biological Sciences, Hussain Ebrahim Jamal Research Institute of Chemistry, University of Karachi, Karachi, Pakistan, from August 2014 to August 2015.

### 2.2. Collection and Identification of Plant Material

The plant *Heliotropium strigosum* was collected in September 2014 from the surroundings of the railway ground district Khanewal (Pakistan) and identified by Dr. Muhammad Zafarullah, Assistant professor of Institute of Pure and Applied Biology, Bahauddin Zakariya University, Multan, Pakistan. A voucher no. “Stewart 591” was assigned to the specimen and preserved in the University herbarium.

### 2.3. Preparation of Dichloromethane Extract

Drying of the whole plant material was achieved by placing it under shade on old newspapers for 25 days to accomplish the process of effective extraction. When the plant material was completely dried, it was made into a coarse powder by crushing in the grinding mill. The extraction of this powdered plant material was carried out by the process of simple maceration. About 600 g of measured powdered material was put into the extraction bottle and a known volume of dichloromethane (3 × 1.5 L) was added into the bottle. For the purpose of maximum possible extraction, the mixture was continuously shaken after every 15 min for 3 to 4 h and then make it homogenized by the method of ultra-sonication. After 24 h, this mixture was filtered off. Repeated the same procedure thrice with dichloromethane. After the third collection, the dichloromethane extract was concentrated separately with the help of a rotary evaporator. This was done under reduced pressure. Then the dichloromethane extract was collected in the separated sample bottle and assigned the code as HSWPD (Heliotropium strigosum whole plant dichloromethane extract). The powdered plant material yielded 5.15 g of crude dichloromethane extract which is approximately 0.85% of total dry weight.

### 2.4. Antimicrobial Assays

#### 2.4.1. Antibacterial Assay―Microplate Alamar Blue Assay (MABA)

The dichloromethane extract of *H. strigosum* was tested for significant antibacterial activity by using microplate alamar blue assay (MABA) (Invitrogen Corporation, San Diego, CA, USA). Strains of four pathogenic bacteria such as *Staphylococcus aureus* (NCTC 6571), *Shigella flexneri*, *Pseudomonas aeruginosa* (ATCC 10145) and *Escherichia coli* (NCTC 10418) were used in this assay. Mueller Hinton medium was prepared in a separate petri dish by following the specifications and guidelines given by the manufacturer (Sigma-Aldrich, St. Louis, MO, USA) and adjusted the pH to 6.6–7.3 normally at 25 °C. In this method, the tested micro-organisms were cultured in Mueller Hinton medium. Then the adjustment of turbidity index of inoculums up to 0.5 McFarland was done. We prepared the standard solution of 1g dichloromethane crude extract in 1 mL sterile dimethyl sulfoxide (DMSO) and distributed the above prepared media into the wells. This work was repeated thrice. All the tested micro-organisms were also placed into the wells. We made sure that the control wells did not contain any testing organism. The volume of well plate 96 was settled up to the level of 200 µL. At the end, added 5 × 10^6^ cells into all the control and testing wells. Sealed all the plates with the help of paraffin. We placed these plates into the incubator at 37 °C for at least 18–20 h without shaking. After 20 h, we added 5 µL alamar blue dye into every well and shook gently at the speed of 80 revolutions per minute for 2–3 h by using the shaking incubator at 37 °C. Plates were covered with foil in shaking incubator. If the color of alamar blue dye was changed from blue to pink, it confirmed the growth of bacteria. Finally, the absorbance at the wavelength of 570 nm–600 nm was recorded with the help of Elisa reader [14].

#### 2.4.2. Antifungal Assay―Agar Tube Dilution Method

The dichloromethane extract of whole plant of *H. strigosum* was tested for certain antifungal activity against six fungal strains such as *Trychophyton rubrum*, *Aspergillus niger*, *Fusarium solani*, *Candida albicans* and *Microsporum canis* by using agar tube dilution assay. We took 24 mg of dichloromethane crude extract and mixed in 1 mL sterile dimethyl sulfoxide (DMSO) to prepare its stock solution. For the preparation of Sabouraud dextrose agar (SDA) medium, we dissolved 32.5 g of Sabouraud glucose (2%) or maltose agar in 500 mL of distilled water. Adjusted the pH of this medium up to 5.5–5.6. Steaming of this media was done to dissolve all the suitable contents and added an appropriate volume (4 mL) into the test tubes having screw caps. These tubes were placed into the autoclave for 15 min at the temperature of 121 °C. After this, these tubes were placed at the temperature of 50 °C to achieve the effective cooling. Then, we loaded the non-solidified agar media by using pipette with 66.6 µL of tested sample taken from the stock solution. At room temperature, these tubes were placed for solidification in a slanting position. In every tube, a piece of fungus obtained from the seven day old fungus culture with the diameter of 4 mm was inoculated. All these tubes containing culture of fungus were placed in the incubator at the optimum temperature of 27 °C–29 °C. These culture containing tubes were allowed to grow for 3–7 days. This culture was observed twice a week during the period of incubation. When the incubation for 3–7 days was completed, the tube in which the growth of fungus culture was not visible was taken for the measurement of MIC value of the tested sample which is expressed in µg/mL [15].

### 2.5. Cytotoxic Assay―Brine Shrimp Lethality Bioassay

We stored the brine shrimp (*Artemia salina* Leach) eggs usually at the very low temperature of 4 °C. When filtration of brine solution was achieved, the hatching tray was filled half with this solution. Then we sprinkled eggs (50 mg) on the hatching try and incubated at the temperature of 37 °C. Then, we prepared the stock solution by taking 20 mg of crude dichloromethane extract and dissolved it in 2 mL of sterile dimethyl sulfoxide. From this stock solution, we transferred 5 µL, 50 µL and 500 µL into three separate glass vials at the concentrations of (10, 100 and 1000) µg/mL by using micro-pipette. We placed these solvent containing vials over night for the evaporation of solvent. After two days of hatching process, we placed 10 larvae individually in the vials by using Pasteur pipette. We made the final volume of solvent up to 5 mL with the addition of sea water. These vials were placed into the incubator at the temperature of 25–27 °C for 24 h beneath the illumination. We took an extra two vials, one of which had the standard cytotoxic drug (Etoposide) served as positive control and the other vials in which respective solvent was added and served as negative control. Larvae were observed in each vial after 24 h. The number of survivors should be determined. Finney computerized system (Probit analysis) was used to analyze the data for the determination of LD_50_ values with 95% confidence intervals. Shrimps can be used 48–72 h after the initiation of hatching. After 72 h they should be discarded [15,16,17].

### 2.6. Phytotoxic Assay―Lemna Bioassay for Phytotoxicity

We prepared the inorganic E-medium (stock solution) by mixing appropriate inorganic constituents [15] into 1 liter of distilled water. The pH of E-medium was adjusted by adding potassium hydroxide pellets up to 6–7. To prepare the working E-medium, 100 mL of this stock solution was taken and dissolved it in 900 mL of distilled water. Then, we prepared the solution of tested crude extract by dissolving 30 mg of crude extract in 1.5 mL of ethyl alcohol. Three flasks were taken and pipetted 10 µL, 100 µL and 1000 µL into these flasks from concentration solutions of (10, 100 and 1000) µg/mL. Placed these solvent containing flasks over night for the evaporation of solvent. In each flask, added 20 mL stock solution of working E-medium along with the addition of 2 to 3 fronds (total fronds used 20) from the rosette of the plant *Lemna minor*. Two supplemented flasks, one with the standard drug (Paraquat) and other with the E-medium were served as positive and negative control respectively. We placed all these flasks into the growth chamber for 7 days by maintaining the temperature at 28 °C along with the light intensity of 9000 lux and relative humidity of 56% ± 10%. When the incubation period was completed, counted and verified the number of fronds of each flask on the 7th day [18,19]. The results which were analyzed as growth inhibition (%), compared with reference drug to negative control were given as shown below.

% inhibition of growth = 100 − (No. of fronds in tested sample/No. of fronds in negative control) × 100

### 2.7. Antioxidant Assay―DPPH (2,2-Diphenyl-1-Picrylhydrazl) Radical Scavenging Assay

We dissolved the weighed amount of tested sample in a suitable volume of pure dimethyl sulfoxide. Then we prepared the 300 µL solution of DPPH (2,2-diphenyl-1-picrylhydrazl) by using appropriate volume of pure ethyl alcohol. About 5 µL of the tested sample solution was added into the 96-well plate and measured the absorbance at the wavelength of 515 nm. After this, to each well was added 95 µL solution of DPPH. We then incubated the 96 well plate at the temperature of 37 °C for the period of 30 min. We covered this plate with paraffin so that the solvent evaporation must be avoided. The pure dimethyl sulfoxide was served as control. By using a micro-plate reader, final absorbance at the wavelength of 515 nm was recorded. Percentage of radical scavenging activity (%RSA) can be determined by the following equation [20].

% RSA = 100 − (Original dose of tested sample/Original dose of control) × 100

## 3. Results

### 3.1. Antibacterial Activity

In the present study, antibacterial potential of crude dichloromethane extract was examined. The percent (%) inhibition showed by sample extract against various tested bacteria are specified in the Table 1. The results of preliminary antibacterial activity demonstrated that the dichloromethane crude extract of *H. strigosum* showed inhibition up to 16% against *S. aureus* while against *S. flexneri*, and showed an inhibition of 3% respectively at the concentration used, which is 150 µg/mL. The pathogens *E. coli* and *P. aeruginosa* showed no inhibition against the tested sample. Thus, crude dichloromethane extract showed non-significant antibacterial activity against all the four tested bacterial strains when compared with the reference drug used, which was Ofloxacin (0.25 µg/mL).

### 3.2. Antifungal Activity

The dichloromethane extract of the whole plant of *H. strigosum* was tested for significant antifungal activity. The results shown by tested crude extract against different fungal strains are given in Table 2. The results revealed that none of the tested fungal strains showed any kind of inhibition against the tested sample. So, the dichloromethane extract was found to be inactive against all the tested fungus species at the concentration of the sample used (400 µg/mL).

### 3.3. Cytotoxic Activity

Brine shrimp lethality bioassay was used for cytotoxic screening of dichloromethane extract of the whole plant of *H. strigosum*. The consequences of dichloromethane screening are expressed in Table 3. The results showed that, at the concentration of 10 µg/mL and 100 µg/mL, the number of dead shrimps was only one. However, when the concentration of the tested sample increased up to 1000 µg/mL, the number of dead shrimps was 23 and only 7 shrimps survived. Thus, from the above revealed data, an LD_50_ of 462 µg/mL was calculated as compared with the reference agent (etoposide) that resulted in an LD_50_ of 7.46 µg/mL.

### 3.4. Phytotoxic Activity

The phytotoxic potential of dichloromethane extract of whole plant of *H. strigosum* was studied by using *Lemna minor* phytotoxicity bioassay. The growth inhibition (%) shown by the tested crude extract is given in Table 4. The results revealed that the tested sample showed percent growth inhibition up to 35% and 40% at the concentration of 10 and 100 µg/mL but at the concentration of 1000 µg/mL, it showed 50% growth inhibition. So, the dichloromethane extract exhibited low phytotoxic activity at the concentrations of 10 and 100 µg/mL but showed moderate activity at the highest tested concentration that was 1000 µg/mL when compared with the standard drug (Paraquat) which inhibited the growth of *L. minor* at the concentration of 0.015 µg/mL.

### 3.5. Antioxidant Activity

DPPH radical scavenging assay was used to determine the significant antioxidant activity of dichloromethane extract of the whole plant of *H. strigosum*. The radical scavenging activity exhibited by the tested dichloromethane extract is shown in Table 5. The results demonstrated that the crude extract showed non-significant antioxidant potential with very low radical scavenging activity up to 13% at the tested concentration that was 500 µM when compared with N-acetyl cysteine (standard drug) which exhibited significant radical scavenging activity up to 96% with IC_50_ of 111.44 ± 0.7 µM respectively at the same concentration.

## 4. Discussion

The history of human beings has revealed that, for the past 60,000 years, plants have been used as a source of treating various ailments in different civilizations of the world [21]. Due to the increased resistance of microbes worldwide, scientists are always looking for the development of newer antibacterial agents [22]. Medicinal plants are considered as a source in the discovery and advancement of new pharmaceuticals which are effective in the management of different diseases [23,24]. In our study, the dichloromethane extract of whole plant material of *H. strigosum* showed non-significant antibacterial activity against *S. aureus*, *S. flexneri*, *E. coli* and *P. aeruginosa*. The results of our studies are quite comparable with the antibacterial activity revealed by other species of genus *Heliotropium* and some other plant species of different families. The petroleum ether and chloroform fractions of ethanolic extract of *H. subulatum* showed strong antibacterial activity against *E. coli*, *S. aureus*, *Streptococcus pneumonia* and *Bacillus subtilis*. That strong antibacterial activity might be due to the purification of five pyrrolizidine alkaloids from this plant [25]. The methaolic extract of aerial parts of *H. indicum* has broad spectrum antibacterial activity against *S. aureus*, *S. pneumonia*, *Salmonella typhi*, *E. coli* and *Klebsiella pneumonia* [26]. The antibacterial significance of plant extracts is attributed mainly to the presence of terpenoids [27]. The sterols and triterpenoids such as β-sitosterol, stigmasterol, β-amyrin, friedelan-β-ol, cycloartenone, β-amyrin acetate and friedelin isolated from ethanol extract of the whole plant of *H. ellipticum* exhibited strong antibacterial activity against *E. coli*, *S. aureus* and *K. pneumonia* [28]. Filifolinol, one of the geranyl aromatic derivatives isolated from *H. sclerocarpum* and *H. filifolium* also showed significant antibacterial activity against *S. aureus*, *Bacillus cereus*, *B. subtilis* and *Micrococcus luteus* [29,30]. Another reported antibacterial potential was shown by the group of new 3 *H*-Spiro[1-benzofuran-2,1′-cyclohexane] derivatives such as 3′-hydroxy-2′,2′,6′-trimethyl-3*H*-spiro[1-benzofuran-2,1′-cyclohexane]-5-carboxylic acid, methyl 3′-acetyloxy-2′,2′,6′-trimethyl-3*H*-spiro[1-benzofuran-2,1′-cyclohexane]-5-carboxylate, methyl 3′-isopentanoyloxy-2′,2′,6′-trimethyl-3*H*-spiro[1-benzofuran-2,1′-cyclohexane]-5-carboxylate, and methyl 3′-benzoyloxy-2’,2′,6′-trimethyl-3*H*-spiro[1-benzofuran-2,1′-cyclohexane]-5-carboxylate isolated from the dichloromethane cuticle extract of *H. filifolium* against several gram positive and gram negative bacteria [31]. In addition to the genus *Heliotropium*, other plant species also exhibited antibacterial potential. The ethanolic extracts of *Curcuma longa* and *Alpinia galangal* displayed weak antibacterial activity against *S. aureus* and *S. typhi* and showed no inhibition against *B. subtilis*, *E. coli*, *P. aeruginosa* and *S. flexneri* [32]. Thus, from the above contributions, researchers have come to know that the identification, isolation and purification of different groups of organic compounds, mainly pyrrolizidine alkaloids, terpenoids and flavonoids, from *H. strigosum* should reveal that this plant has a perceptible future role in the field of antibacterial medicinal agents.

Different pathogenic fungi cause systemic infections of the skin, lungs, liver, mouth and blood [33,34,35]. Fungal attacks especially infect the skin and cause severe infections such as athlete’s foot, tinea cruris and numerous others [36]. Only 10 antifungal drugs are permitted for the therapeutic management of invasive systemic fungal infections by the Food and Drug Administration (FDA) authority in United States of America [37]. Patients who have had pediatric lung transplantation are at serious risk of pulmonary fungal infection [33]. In the present study, the dichloromethane extract of whole plant material of *H. strigosum* demonstrated no antifungal activity and was found to be inactive against all the tested fungal strains. These results exposed the antifungal principles in a consistent manner, as established by different plant species of the family *Boraginaceae* and some other families. The methanolic extract of *Echium rauwolfii* and *E. horridum* showed no antifungal activity against *Aspergillus flavus* but were found to have weak activity against *C. albicans* [38]. The dichloromethane extract of *Cordia curassavica* [39] and *Cordia linnael* [40] showed equal antifungal activity to some extent against both *C. albicans* and *Clasdosporium cucumerinum*. The benzene extract of *Trichodesma amplexicaule* remained inactive against *Aspergillus niger* and *A. flavus* followed by chloroform extract with very low activity against these micro-organisms [41]. The ethyl acetate and n-butanol fractions of methanolic extract of *Onosma griffithii* reported no antifungal activity [42]. The ethyl alcohol and aqueous extracts of *Colendia procumbens* never showed antifungal activity against *C. albicans* [43]. No antifungal activity was displayed by the methanolic and aqueous extracts of *Trichodesma zeylanicum* and *Anchusa italic* [44]. Apart from the above consequences, different biological extracts and isolated bioactive constituents, mainly pyrrolizidine alkaloids and terpenoids from different *Heliotropium* species such as *H. indicum* [45], *H. ellipticum* [28,46] and *H. marifolium* [47], demonstrated significant antifungal activity against various pathogenic fungi. Our study reports for the first time the dichloromethane screening of *H. strigosum* for antifungal potential.

Due to the life threatening effects of cancer, it is known as a critical factors that increases the mortality rate worldwide. Cancer of the liver, lung, colon and stomach contribute to a major proportion of mortality cases all over the world [48]. In the present research work, the dichloromethane extract of *H. strigosum* shows positive cytotoxic activity with an LD_50_ of 462 µg/mL. The cytotoxic potential of this plant is extremely compatible with the previously reported developments in the field of antitumor drug discovery. The ethanolic, dichloromethane and n-hexane extracts of *H. subulatum* [49] and the methanolic extract of *H. indicum* [50] showed significant antineoplastic activity in the dose dependent manner. The ethanolic extracts of *Curcuma longa* and *Alpinia galangal* revealed strong cytotoxic potential with the LD_50_ of 33 µg/mL and 109 µg/mL, respectively [32]. The occurrence of cytotoxic activity was mainly due to the presence of different classes of organic compounds such as phenolic compounds (polyphenols), catechins and flavonoid constituents. A large number of polyphenols and flavonoids have been purified and isolated from different parts of *Bruguiera gymnorrhiza*, *Blumea lacera*, *Aegiceras corniculatum*, *Hygrophila auriculata* and *Hibiscus tiliaceous,* which might be responsible for their cytotoxic activity. In 2007, a scientific report from Bangladesh examined 32 extracts of 16 different Bangladehsi plant species for their cytotoxic potential. Among these tested extracts, the aqueous extracts possessed very low cytotoxic potential. This low antitumor potential of aqueous extracts is of great therapeutic significance because traditionally they are used in the medication of various ailments instead of cancer [51]. Another informative description of cytotoxic potential was reported in Brazil in which 60 species of different medicinal plants were screened to examine the cytotoxic activity. Only 10% of plant species showed better cytotoxicity with ED_50_ less than 1000 ppm [16]. In most of the reported statistical data about the cytotoxic potential of different medicinal plants, scientists used brine shrimp lethality bioassay instead of other scientific approaches because a progressive correlation existed between the toxicity of brine shrimp and human nasopharyngeal carcinoma [52].

In the developing countries, weeds are considered an important factor for environmental protection. Approximately 30,000 different species of weeds are present in the world, out of which a reported 1800 cause the loss of 9.7% of crop yields [53]. The presence of weeds reduced the agricultural productivity of crops that lead to massive economical loss among different regions of the world. In a recent study, the dichloromethane extract of *H. strigosum* demonstrated moderate phytotoxic activity at the higher tested concentration (1000 µg/mL) but showed low phytotoxic activity at the concentration of 10 and 100 µg/mL with the growth inhibition of 35% and 40%. The phytotoxic significances of this plant are followed by some other species of genus *Heliotropium* such as *H. dasycarpum* whose methanol and dichloromethane extract showed 100% inhibitory effect at the concentration of 1000 µg/mL, respectively [54]. Similar phytotoxic measurements were shown by ethanolic extract of *Curcuma longa* and *Alpinia galangal* [32]. The methanolic extract of *polygonatum verticillatum* exhibited significant phytotoxic activity at the tested doses of 5, 50 and 500 µg/mL [55]. A few scientific descriptions of allelopathic approaches of *H. indicum* [56] and *Chrysanthemum morifolium* [57] were also considered. The reduction of crops quantity and quality is given serious attention by the scientists who focus upon the discovery and development of newer weedicides because chemically synthetic weedicides cause harmful adverse effects, mainly the expansion of weedicide resistant populations, lowering the water and soil consumption and resulting in damaging effects on non-targeted organisms [53].

The destructive effects of free radicals can be prevented by using different organic and inorganic substances having low molecular weight. Some of the commonly used antioxidative agents are tocopherols, copper, vitamin C, zinc, thiols, iron and manganese [58]. The recent scientific studies also exposed the beneficial preventive effects of antioxidants in the treatment of cardiovascular diseases, ocular damage and certain type of cancers [59]. The consequences of our study shows that the dichloromethane extract of *H. strigosum* demonstrated very low radical scavenging activity, only up to 13%. Therefore, no antioxidant activity was shown by the tested extract and it was found to be inactive. However, some other species of genus *Heliotropium* showed excellent radical scavenging activity because of the presence of flavonoids. Flavonoids are the largest group of naturally occurring phenolic compounds and possessed some chemical and biological properties which are very helpful in the prevention of free radicals formation. Flavonoids are found to be ubiquitous in most of the plants growing in extreme conditions [60]. A new compound belonging to the group of flavanones named as Naringenin was identified and isolated from the resinous exudate of dichloromethane extract of *H. sclerocarpum* which displayed outstanding free radical scavenging activity [30]. In 2009, from the resinous exudates of dichloromethane extract of *H. taltalense*, three flavonoids, namely Naringenin, 3-*O*-methylgalangin and 7-*O*-methyleriodictiol were isolated and exhibited significant antioxidant activity [61]. The isolation of three new flavonoids, namely, 5,3′-dihydroxy-7,4′-dimethoxyflavanone, 5,4′-dihydroxy-7-methoxyflavanone and 4′-acetyl-5-hydroxy-7-methoxyflavanone from *H. glutinosum* was also reported [60]. From *H. sinuatum*, eight previously reported flavonoids along with one new compound 4-(3′,5′-dihydroxynonadecyl) phenol were isolated, which confirmed the antioxidant behavior of this plant [62]. All the above previous scientific reports confirmed that all these plants showed exceptional antioxidant potential. This gives scientists a new approach to identify, purify and isolate the flavonoids and other phenolic compounds from *H. strigosum* which lead towards the synthesis of new antioxidant agents in the world of medicines.

## 5. Conclusions

The dichloromethane screening of *Heliotropium strigosum* was done for the first time. The crude extract of this plant showed low antibacterial activity against two bacterial stains, one is gram positive and other is gram negative. There was no antifungal activity shown by the tested crude extract. The dichloromethane extract exhibited positive cytotoxic and moderate phytotoxic potential at the highest tested concentrations. No antioxidant behavior was exposed by this plant. In conclusion, scientists and pharmacologists should pay serious attention to the screening of this plant by using some other scientific bioassay methodologies which might serve as a source for the identification, purification and isolation of beneficial bioactive constituents that seems to be helpful in the synthesis of new therapeutic agents of desired interest. Therefore, in the future, *Heliotropium strigosum* will be used globally as a source of safer phytomedicines.

## Figures and Tables

**Table 1:** Results of preliminary antibacterial assay of *H. strigosum*.

Name of Bacteria	Percent (%) Inhibition of Tested Sample	Percent (%) Inhibition of Standard Drug (Ofloxacin)
*S. aureus*	16	92.35
*E. coli*	0	90.20
*P. aeruginosa*	0	89.35
*S. flexneri*	3	91.40

Concentration of tested sample used = 150 µg/mL; Concentration of standard drug used = 0.25 µg/mL; Positive control = Ofloxacin (standard antibiotic); Negative control = DMSO (Dimethyl sulfoxide); Percent inhibition activity, 0–39 = Low (non-significant); 40–59 = moderate; 60–69 = Good; above 70 = Significant.

**Table 2:** Results of antifungal assay of *H. strigosum*.

Name of Fungus	Linear Growth (mm)		% Inhibition (Tested Sample)	Standard Drug	MIC (µg/mL)
	Sample	Control			
*T. rubrum*	100	100	0	Miconazole	97.8
*A. niger*	100	100	0	Amphotericin B	20.70
*F. solani*	100	100	0	Miconazole	73.50
*C. albicans*	100	100	0	Miconazole	113.1
*M. canis*	100	100	0	Miconazole	98.1

MIC = Minimum inhibitory concentration; Concentration of tested sample used = 400 µg/mL; Percent inhibition activity, 0–39 = Low (non-significant); 40–59 = moderate; 60–69 = Good; above 70 = Significant; Positive control = Miconazole and Amphotericin B (*A. niger*); Negative control = DMSO (Dimethyl sulfoxide).

**Table 3:** Results of brine shrimp lethality bioassay of *H. strigosum*.

Dose of Tested Sample (µg/mL)	No. of Shrimps	No. of Survivors	LD_50_ (µg/mL)	Standard Drug	LD_50_ (µg/mL)
10	30	29	462	Etoposide	7.46
100	30	29			
1000	30	7			

Positive control = Etoposide; Negative control = DMSO (Dimethyl sulfoxide); No. of replicates = 3; Incubation conditions = 28 ± 1 °C.

**Table 4:** Results of phytotoxicity assay of *H. strigosum*.

Name of Plant	Concentration of Tested Sample (µg/mL)	No. of Fronds		% Growth Inhibition	Concentration of Standard Drug (Paraquat) (µg/mL)
		Sample	Control		
*Lemna minor*	10	13	20	35	0.015
	100	12		40	
	1000	10		50	

Positive control = Paraquat; Negative control = Volatile solvent (ethanol); Incubation conditions = 28 ± 1 °C.

**Table 5:** Results of diphenyl picryl hydrazine (DPPH) radical scavenging assay of *H. strigosum*.

Concentration of Tested Sample (µM)	% RSA (Radical Scavenging Activity)	IC_50_ ± SEM (µM)
500 ^a^	13	>500
500 ^b^	96	111.44 ± 0.7

^a^ = Dichloromethane extract; ^b^ = N-acetyl cysteine (Standard drug & Positive control); Negative control = DMSO (Dimethyl sulfoxide); µM = Micro-molar (10^−3^ mol/m^3^); SEM = Standard error mean; Data is expressed as mean ± SEM of three independent readings.
